# New machine learning model predicts who may benefit most from COVID-19 vaccination

**DOI:** 10.1038/s41746-021-00425-4

**Published:** 2021-03-26

**Authors:** Leia Wedlund, Joseph Kvedar

**Affiliations:** 1 Harvard Medical School, Boston, MA USA; 2 Mass General Brigham, Boston, MA USA

**Keywords:** Prognosis, Preventive medicine

As the COVID-19 vaccine roll-out continues, a vigorous debate rages over who should receive the limited available doses. While the CDC and state governments struggle to prioritize vaccine recipients based on occupation, demographics, and comorbidities, researchers at Mass General Brigham have developed a precise new tool to identify which individuals may benefit most from vaccination.

A new machine learning model, described in *npj Digital Medicine* this February by Estiri et al.^1^, uses electronic health records to accurately predict an individual’s probability of death if they are infected with COVID-19. While many models created over the past year have predicted COVID-19 mortality based on vitals and labs collected during hospitalization^2,3^, this one provides a more widely applicable tool that allows clinicians to predict which currently uninfected individuals might derive the greatest benefit from vaccination.

Until this point, attempts to identify and vaccinate the most vulnerable have relied on broad demographic categories (e.g.: age >65, obesity, history of cancer). However, these groupings describe heterogeneous populations, and sorting people in this way cannot guarantee that those most at risk truly receive the highest priority. The work of Estiri et al. represents a significant breakthrough because it is the first step towards a maximally precise resource-allocation system that could give individuals priority based on their unique health history and risk of mortality.

This work has implications far beyond the distribution of COVID-19 vaccines. With hospitals worldwide facing finite resources, such a model could help allocate therapies and equipment to those most at-risk, maximizing survival. We have only scratched the surface of what is possible with an ever-growing electronic health record and increasingly accurate predictive analytics.
